# Chitosan-Enriched Salicylic Acid Nanoparticles Enhanced Anthocyanin Content in Grape (*Vitis vinifera* L. cv. Red Sultana) Berries

**DOI:** 10.3390/polym14163349

**Published:** 2022-08-17

**Authors:** Naser Khalili, Mehdi Oraei, Gholamreza Gohari, Sima Panahirad, Hassan Nourafcan, Christophe Hano

**Affiliations:** 1 Department of Horticultural Sciences, Faculty of Agriculture, Miyaneh Branch, Islamic Azad University, Miyaneh 5315836511, Iran; 2 Department of Horticultural Sciences, Faculty of Agriculture, University of Maragheh, Maragheh 5518183111, Iran; 3 Department of Agricultural Sciences, Biotechnology and Food Science, Cyprus University of Technology Limassol, Limassol 3036, Cyprus; 4 Department of Horticultural Sciences, Faculty of Agriculture, University of Tabriz, Tabriz 5166616471, Iran; 5 Laboratoire de Biologie des Ligneux et des Grandes Cultures, INRAE USC1328, Campus Eure et Loir, Orleans University, 28000 Chartres, France

**Keywords:** antioxidant, fruit quality, grape, nanocomposite, phenolic compounds

## Abstract

Given the effects of salicylic acid (SA) on enhancing the phenolic compounds, flavonoids, and especially anthocyanins at higher doses in grapes as well as some toxic effects of SA at higher doses, the use of nano-carriers and nano-forms could assist SA in enhancing the accumulation of these compounds while reducing its toxic activity. Chitosan (CTS) has gained attention as a safe transporter and control releaser for a variety of chemicals, particularly in the agriculture industry. In this regard, the nano-form combination of SA and CTS (CTS-SA NPs) could boost the effectiveness of SA, particularly at lower dosages. Therefore, in the present study, SA (10, 20 mM), CTS (0.1%), and CTS-SA NPs (10, 20 mM) were applied on grape (*Vitis vinifera* L.) berries cv. Red Sultana at the pre-véraison stage to evaluate their actions on phenolic compounds, particularly anthocyanins. The CTS-SA NPs treatments provided the highest results in terms of the total phenolic compounds, flavonoids (10 mM), anthocyanins (in particular oenin, the main anthocyanin of red grapes) (10 and 20 mM), and PAL enzyme activity (20 mM). In conclusion, the CTS-SA NPs could be applied as a potential effective elicitor for phenolics, particularly anthocyanin enhancement of grape berries at pre- véraison stage with synergistic effects between SA and CTS in nano-forms predominantly at lower doses.

## 1. Introduction

Grape berries comprise various phenolic compounds and anthocyanins, particularly in red and pink ones, where important phytochemicals and secondary natural metabolites contribute significantly to their quality, appearance, taste as well as several other biological activities and health-promoting benefits [1,2,3,4]. Phenolic compounds have various functions in plants s potential roles as effective antioxidants (i.e., direct (e.g., free-radical scavenging ability) and indirect (e.g., by stimulating activity of antioxidant enzymes) impacts) [5], normal growth and development, pigmentation, astringency, stabilizing membranes, hindering the diffusion of free radicals, retarding the oxidative degradation of lipids, restricting peroxidative reaction and defense against infections and injuries [1,6,7,8]. Furthermore, most of them have antimicrobial and UV-absorbing activities [6] next to various nutritional and healthy properties [9,10]. They are primarily synthetized from cinnamic acid by the action of the phenylalanine ammonia lyase (PAL) enzyme [2]. Grape phenolic compounds are affected by various factors, most importantly variety [11]. Anthocyanins, as derivatives of phenolic compounds, are natural water-soluble pigments responsible for the color of various red-purple fruits, vegetables, and flowers [12,13]. They are produced by the secondary metabolism of plants with relevant roles similar to phenolics [12]. Anthocyanins act in anti-aging, as a suppresser of cancer tumors, blood lipid reducer, and a liver protector, consequently with a variety of notable health-promoting effects [14]. Anthocyanin content enhances during fruit ripening, starting at the véraison stage, announced as the end of the developmental stage and beginning of fruit ripening in the berries, in line with some developmental changes such as the loss of skin chlorophyll and the accumulation of sugars in fruits. After the véraison, the content of anthocyanins increases in fruit skin, touches a top in three or four weeks after the stage, and then remains constant until the harvest or in some cases reduced. Furthermore, grape anthocyanins are plant-specific and are produced in phenylpropanoid metabolism and then principally lead to color variation in the berries and their products, presenting variable amounts according to the cultivar. Malvidin-3-glucoside (M3G) (oenin) and its derivatives are primary anthocyanins in red grapes [2]. Therefore, anthocyanin studies have gained more attention over the decades.

Salicylic acid (SA) is an endogenous growth regulator or hormone with regulatory signaling effects in plant physiological processes found in many plant tissues [2,10,15]. SA plays roles in cell growth, development and senescence, respiration, stomatal movement, photomorphogenesis, seed germination [16], defense responses and plant stress resistance [10,15], plant systemic acquired resistance (SAR) [16], and is involved in some signal transduction systems to induce particular enzymes [17]. Moreover, SA increases secondary metabolite production (e.g., phenolic compounds) in plants [2,10,15,18,19,20,21] by increasing PAL activity [2,17]. SA could induce the transcription of the PAL gene in grape berries [22], consequently with an elicitor-like impact on these compounds [2]. Several studies have reported positive effects of SA on the regulation and enhancement of phenolic compounds and anthocyanins [2,10,23]. SA also regulates the protective enzymes (e.g., SOD, POD, APX, GP) [1,15,17]. Frequently, the positive effects of SA occur at lower concentrations while higher levels act antagonistically, confirmed in grape (*Vitis vinifera* cv. ‘Sultana’) by increasing the H_2_O_2_ concentration as a reactive oxygen species (ROS) in tissues [15]. To deal with this challenge, exploiting novel delivery systems, specifically based on nanotechnology that have lately emerged, would enhance the availability of loads such as SA at lower doses with more efficient transportation and release [24].

Chitosan (CTS) is a biocompatible polysaccharide with key affirmative impacts on plant growth and development (e.g., enhancing chlorophyll content and nutrient uptake) [1,24,25] and with encouraging applications, mostly in various delivery systems (e.g., plant growth promoters, fertilizers, genetic materials, pesticides, and herbicides) in the agricultural sector [1,24,26]. In addition, CTS has been demonstrated to have elicitor activity [27,28], particularly in the content of phenolic compounds, anthocyanins and the antioxidant power of grape [29,30], broccoli [31], strawberry [32], and raspberry [33]. CTS also enhanced PAL activity [30,33]. CTS could be used as a perfect adsorption matrix and carrier due to its exceptional chemical and biological properties (i.e., polycationicity, biocompatibility, and biodegradability). CTS, as a carrier, initiates the slow and sustained release and superior efficiency of loads [26], leading to load protection from adverse environmental conditions and removing harmful impacts of the load burst release to plant cells. Moreover, a CTS nano-form (CTS NPs) has attained further approval as a carrier [1,24,30,34,35,36] due to its enhanced properties such as improved physical, biochemical, and antimicrobial properties [25]. Furthermore, CTS is a nontoxic compound for humans, making it an attractive component for usage in agricultural sectors, particularly as a carrier, as previously reported for selenium and phenylalanine, thus introducing a bright future ahead, especially for better efficiency and controlled delivery of any load [24,30,35].

Given that SA and CTS have demonstrated encouraging impacts on plant growth, physiological and biochemical parameters, particularly anthocyanins, aside from the capability of CTS for use as a carrier and control releasing matrix, their combination “chitosan-salicylic acid nanoparticles (CTS-SA NPs)” might cause a synergistic effect to increase their effectiveness, principally SA, at lower doses. Therefore, first, CTS-SA NPs were synthesized, characterized, and then applied on grape berries (*Vitis vinifera* L. cv. Red Sultana) with the hypothesis of the NPs positive effect on anthocyanins at lower doses of SA; this could remove the toxicity effect of SA at higher levels through nano-forms and CTS (as a carrier). Oenin, as a special anthocyanin of red grapes, its derivatives, and some other compounds, were examined by the HPLC method to be more accurate in this regard.

## 2. Materials and Methods

### 2.1. Site Description, Plant Materials, Experimental Design and Treatments

A *Vitis vinifera* L. cv. Red Sultana (a main local seedless cultivar with shiny and red-purple skin) vineyard was selected for the experiment during the 2020 growing season. The vineyard was located on a sandy loam soil near Maragheh, Iran (longitude 46°530 E, latitude 37°380 N) with seven-year-old grapevines planted at a spacing of 2.8 by 1.5 m (2380 vines/ha) and received the common cultural practices in the region. The current experiment was performed in a completely randomized block design with four replications in which every experimental unit was comprised of five grapevines; each grapevine contained no less than three clusters with approximately the same size, maturity, and development. The treatments including salicylic acid (SA) (10 and 20 mM), chitosan (CTS) (0.1%), and “chitosan-salicylic acid nanoparticles” (CTS-SA NPs) (10 and 20 mM) were sprayed on clusters entirely at pre-véraison (in which berries are green and hard) stage. Accordingly, 90 clusters were sprayed that were then evaluated for the parameters. The treatments with surfactant TWEEN^®^ 20 were sprayed three times (150 mL per cluster) with a five-day interval in the early morning on the clusters with green and hard berries and few evidence of asynchrony. An equal amount of distilled water plus TWEEN^®^ 20 was sprayed on the untreated plants (control). At harvest, the berries with enough coloration, softening, and development (9.5–10.5 °Brix) were harvested for the assessments. For each measurement, three replicates were included. All materials were obtained from Sigma-Aldrich Corporation (St. Louis, MO, USA).

### 2.2. Chitosan-Salicylic Acid Nanoparticles Characterization

Chitosan-salicylic acid nanoparticles (CTS-SA NPs) were prepared via the same procedure described by Hassanpour et al. [37]. Characterization analysis of the nanoparticles was conducted by field emission scanning electron microscopy (FE-SEM, FEI Quanta 200 F, FEI, USA) and transmission electron microscopy (TEM Philips EM 208S, Eindhoven, The Netherlands). In this regard, the nano-carriers prepared by the freeze-drying method were dried using a vacuum pump.

### 2.3. Fresh and Dry Weights of Berries, Titratable Acidity, Total Soluble Solids and pH

Fifty berries were weighed for fresh weight (FW) and then kept in the oven (70 °C, 72 h) for dry weight (DW) measurements of each replicate and then the mean was used as the FW and DW of a berry of each replicate. Titratable acidity (TA) was quantified through titration with 0.1 N NaOH up to pH 8.2. The TA was expressed as g tartaric acid L^−1^ FW. A refractometer (PR−1; Atago Co., Ltd., Tokyo, Japan) was used to determine the total soluble solids (TSS) of the berries at 20 °C (expressed as %). The TA and TSS were measured through homogenized berry samples (50 berries). A pH meter (Hanna Instruments, Milan, Italy) was used to record the pH of the berries.

### 2.4. Vitamin C, Total Carbohydrates and Carotenoids

The vitamin (vit) C content of the berries was measured through titration with 0.1 N potassium iodide (KI) [38]. Total carbohydrates were determined through adding the ethanolic extract of berries to an anthrone solution (9 mL), vortexed and placed in a water bath (60 min). Finally, after cooling down, the absorbance of the mixtures was recorded at 216 nm [39]. Grape berries (0.5 g) were homogenized with liquid nitrogen and then extracted with acetone (80%). After centrifuging (1500 rpm, 10 min), the absorbance of the supernatants was recorded at 470 nm to determine the carotenoids [40].

### 2.5. Total Phenolic and Flavonoid Content

The berries were mashed, freeze-dried, and then extracted using MeOH/H_2_O/acetic acid (70:29:1, *v*/*v*/*v*) with a 4/1 (*v*/*w*) ratio solvent/sample on a shaker at 300 rpm for 2 h at room temperature. After removing the supernatant, the pellet was re-suspended with 4 mL of the solvent, the previous step repeated, subsequently centrifuged (10,000× *g*, 10 min), and finally, the supernatant was collected and kept frozen at −20 °C until further analysis. All extractions were performed in triplicate [2]. To assay the total phenolic content, the Folin-Ciocalteu reagent method was applied. In short, to a 200 µL extract in a test tube, 1 mL Folin-Ciocalteu reagent and 800 µL Na_2_CO_3_ (7.5%) were added, mixed, left to stand for 30 min, and then the absorption was recorded at 765 nm using a UV–Vis spectrophotometer (Analytik Jena AG, Jena, Germany). The total phenolic content was calculated using a standard curve of gallic acid and expressed as mg gallic acid 100 g^−1^ fresh weight (FW) [24].

After extracting the berries (1 g) with 96% ethanol (4 mL), centrifuging, and collecting the supernatant, to the supernatant (1300 µL), ethanol (96%, 700 µL), aluminum chloride (10%, 100 µL), potassium acetate (100 µL, 1 M), and finally distilled water (2.8 mL) were added and kept at room temperature (30 min). Finally, the solution absorbance was recorded versus a blank at 415 nm and the final flavonoid results were attained through the standard curve obtained by different concentrations of quercetin and expressed as mg quercetin 100 g^−1^ FW [2].

### 2.6. Total Anthocyanin Content and Anthocyanin Analysis

The pH differential method was applied to assay the total anthocyanins. After extracting the berries with 2% HCl in methanol (24 h, in the dark, and at room temperature), the extracts were diluted to an appropriate concentration with potassium chloride buffer (pH 1.0) until the sample absorbance was within the linear range of the spectrophotometer (0–1.2). The spectrophotometer was blanked with distilled water. Two dilutions of each sample were prepared: one with potassium chloride buffer (pH 1.0) and the other with sodium acetate buffer (pH 4.5), the dilutions were allowed to stand for 15 min and their absorbance were recorded at 520 and 700 nm. The concentration of the total monomeric anthocyanins was determined using MW = 528.89 g mol^−1^ and ε = 28,000 L cm^−1^ mol^−1^ and expressed in mg oenin, the main anthocyanins from grapes, 100 g^−1^ FW [41].

The analysis of anthocyanins in the extracts was performed by the HPLC method. After centrifuging (10,000× *g*, 4 °C, 10 min), the juice of the berries (obtained from the whole berry including skin and tissue) were kept frozen at −20 °C until the analysis. The HPLC instrumentation consisted of a Knauer mixing chamber, two Knauer 64 pumps, and a Knauer UV/VIS Detector (Knauer, Berlin, Germany). A C18 column (4.6 mm × 250 mm, 5 µm; Knauer Eurospher I 100-5, Germany), coupled with a pre-column with the same stationary phase, was used. The analytical standard of malvidin-3-O-glucoside (molecular formula C_23_H_25_ClO_12_) was purchased from Polyphenols Laboratories AS (Sandnes, Norway). In addition, formic acid and methanol (HPLC grade) were purchased from Merck (Darmstadt, Germany). The clarified juice (20 µL) was injected onto the HPLC. The elution was carried out at room temperature using 5% formic acid (A) and methanol (B) in a linear gradient from 15 to 35% B until 15 min, followed by isocratic elution until 20 min. The flow rate was 1 mL min^−1^ with the UV/VIS detector at 510 nm. Quantification was based on the external standard method and malvidin-3-O-glucoside and other compounds were identified by a comparison of its retention times with those of the analytical standard reference samples. This procedure was repeated in triplicate [42].

### 2.7. Antioxidant Capacity

The antioxidant capacity was evaluated using the DPPH (2,2-diphenyl-1-picrylhydrazyl) colorimetric method [43] by adding 3.9 mL of DPPH (2.5 × 10^−2^ gL^−1^ in methanol) to 100 µL extract and recording the absorbance at 515 nm and at different time intervals until the reaction reached a plateau (steady state). The below formula was used to calculate the final results expressed as a percentage (%):

% Total antioxidant activity = (A blank − A samp)/(A blank) × 100

### 2.8. PAL Enzyme Activity

To assay the PAL activity, the berries (1 g) were ground in liquid nitrogen and then extracted with PVP (50 mg) and 0.1 M phosphate buffer (pH 7.2, 2 mM EDTA, 4 mM DTT) (2 mL) at 4 °C. After centrifuging the mixture (10,000× *g*, 4 °C, 10 min), the supernatant (200 µL) was used for enzyme activity after incubation with L-phenylalanine (dissolved in 10 mM borate buffer, pH 8.8) (20 mM, 1000 µL), borate buffer (10 mM, pH 8.8, 2000 µL), and distilled water (1000 µL) at room temperature (60 s). Finally, the absorbance of the mixture was recorded at 290 nm and used to calculate the PAL activity [2].

### 2.9. Statistical Analyses

After data collection, the analysis of variance was performed. A homogeneity test of variance was conducted before analyzing the data. Tukey’s post hoc test was used to compare the means. Differences of *p* ≤ 0.05 were considered significant. All statistical analyses were conducted in SPSS version 19 for Windows (IBM SPSS Statistics Release 19.0. 0.1, 2011, SPSS Inc., Chicago, IL, USA).

## 3. Results

### 3.1. Characterization of CTS-SA NPs

**Figure 1:**
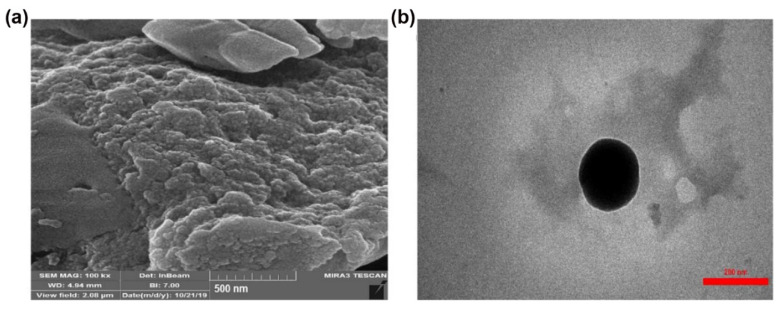
The scanning electron microscopy (SEM) (**a**) and transmission electron microscopy (TEM) (**b**) images of the chitosan-salicylic acid nanoparticles (CTS-SA NPs).

The surface morphology of the CTS-SA NPs was investigated using scanning electron microscopy (SEM). The SEM image showed spherical particles without porosity in Figure 1a for CTS-SA NPs. The transmission electron microscope (TEM) image also showed a spherical shape of CTS-SA NPs with an average size of about 170–180 nm (Figure 1b).

### 3.2. FW and DW of Berries, TA, TSS, TSS/TA Ratio, and pH

Table 1 presents the results obtained for the FW and DW of the berries, TA, TSS, TSS/TA ratio, and pH traits after the application of the SA (10 and 20 mM), CTS NPs (0.1%), and CTS-SA NPs (10 and 20 mM) treatments. In general, all of the applied treatments positively affected the traits through enhancing the FW, DW, TSS, TSS/TA, and pH and reducing the TA compared to the control. CTS-SA NPs at 20 mM acted as the best treatment considering FW and DW; CTS-SA NPs at 10 mM resulted in the highest TSS and CTS-SA NPs at 10 and 20 mM were the best treatments regarding the TSS/TA and pH (Table 1).

### 3.3. Vit C, Total Carbohydrates and Carotenoids

**Figure 2:**
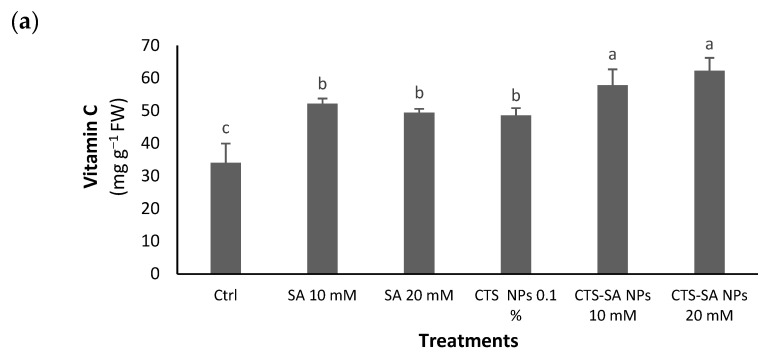

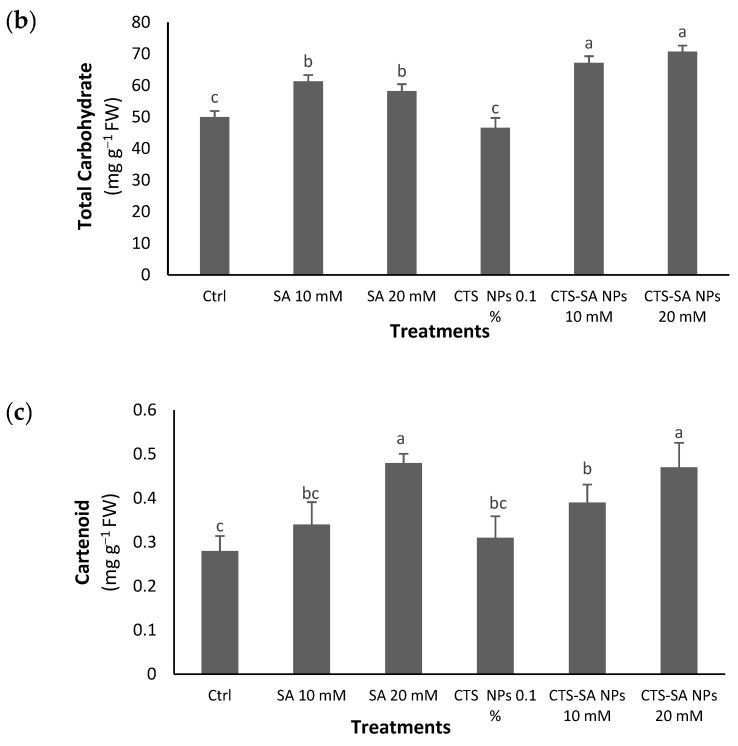
The effect of salicylic acid (SA) (10 and 20 mM), chitosan (CTS) (0.1%), and their combination in nano-form (CTS-SA NPs) treatments on vitamin C (vit C) (**a**), total carbohydrates (**b**), and carotenoids (**c**) of grape (*Vitis vinifera* L.) cv. Red Sultana. Different letters indicate significantly different values at *p ≤* 0.05.

All of the applied treatments increased the vit C (Figure 2a). The total carbohydrates were positively affected by the SA and CTS-SA NP treatments (Figure 2b). Regarding vit C and total carbohydrates, the CTS-SA NP treatment at both applied levels acted as the best, introducing the lower dose better. Concerning the carotenoids, SA 20 mM and both CTS-SA NPs enhanced the content with the best results in SA and CTS-SA both at a 20 mM concentration (Figure 2c).

### 3.4. Total Phenolic Compounds, Flavonoid and Anthocyanin Contents, and Anthocyanin Analysis

**Figure 3:**
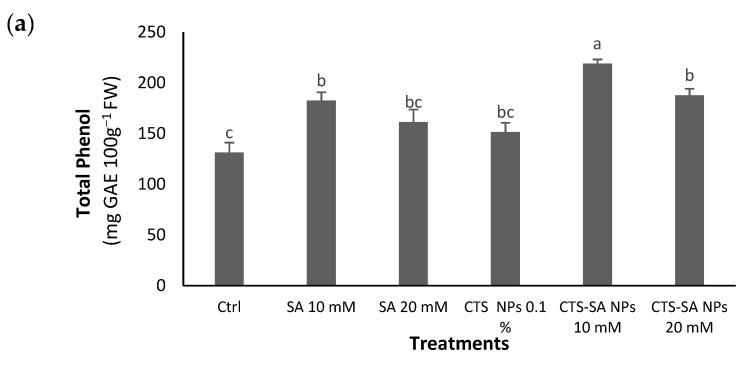

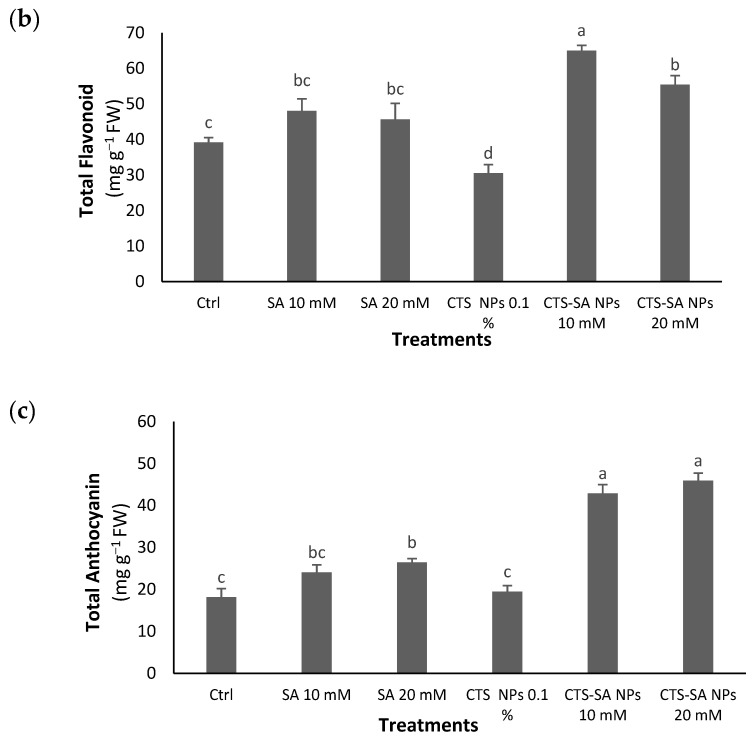
The effect of salicylic acid (SA) (10 and 20 mM), chitosan (CTS) (0.1%), and their combination in nano-form (CTS-SA NPs) treatments on the total phenolic compounds (**a**), flavonoids (**b**), and total anthocyanin (**c**) contents of grape (*Vitis vinifera* L.) cv. Red Sultana. Different letters indicate significantly different values at *p ≤* 0.05.

The SA at a 10 mM concentration and CTS-SA NPs at both levels enhanced the total phenolics compared to the control with the highest content at 10 mM CTS-SA NP treatment (Figure 3a). Considering the flavonoid and anthocyanin contents, only CTS-SA NP treatments enhanced their content; the best results were achieved by 10 mM CTS-SA NPs, and 10 and 20 mM CTS-SA NPs for the flavonoids and anthocyanins, respectively (Figure 3b,c). High-performance liquid chromatography (HPLC) chromatograms of the anthocyanins are presented as data in Table 2.

The main compounds of fresh berries were malvidin-3-O-β-glucoside (oenin), malvidin-3-O-acetylmonoglucoside, malvidin-3-(6-O-p-coumaroyl) monoglucoside, peonidin-3-O-monoglucoside, delphinidin-3-O-monoglucoside, petunidin-3-O-monoglucoside, and cyanidin-3-O-monoglucoside, in that order. Compared to the untreated fruits, anthocyanin accumulation was mostly higher in the treated fruits, especially the CTS-SA NP treatments. To be more accurate, all of the applied treatments including SA at both levels, CTS NPs and CTS-SA NPs at both levels enhanced oenin (as the dominant compound of the red grapes like Red Sultana) and delphinidin-3-O-monoglucoside; SA and CTS-SA NPs at both levels increased cyanidin-3-O-monoglucoside and petunidin-3-O-monoglucoside; and the CTS-SA NP treatments had a positive impact on peonidin-3-O-monoglucoside. CTS-SA NPs at a 20 mM concentration enhanced malvidin-3-O-acetylmonoglucoside while CTS-SA NPs 20 mM, besides SA 10 mM increased malvidin-3-(6-O-p-coumaroyl) monoglucoside (Table 2). Considering all compounds, particularly oenin, as the most important one and the case study, CTS-SA NPs, especially at the lower dose, could be considered as the best treatment to enhance the value almost twice more than the control.

### 3.5. Antioxidant Capacity Based on DPPH and PAL Enzyme Activity

**Figure 4:**
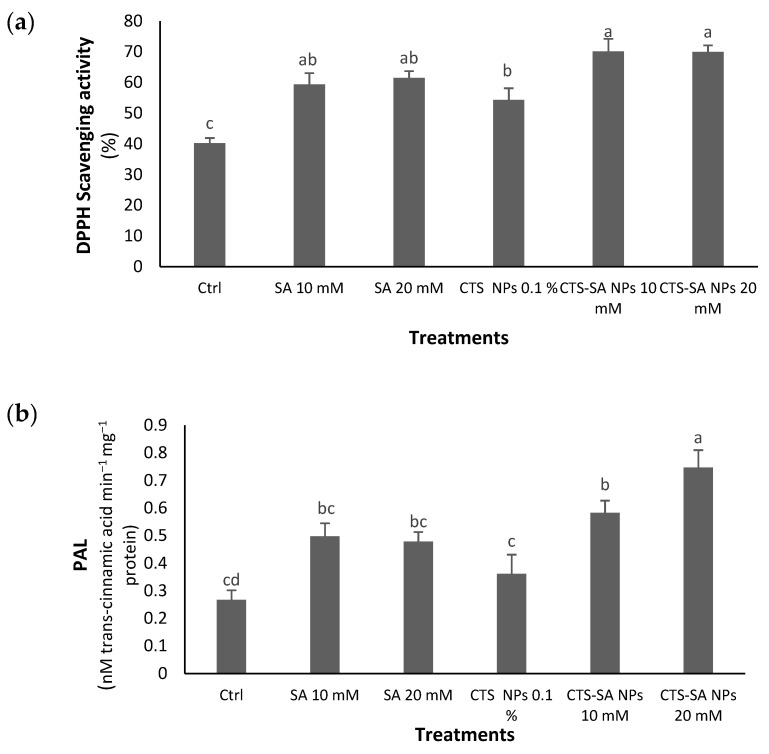
The effect of salicylic acid (SA) (10 and 20 mM), chitosan (CTS) (0.1%), and their combination in nano-form (CTS-SA NPs) treatments on the DPPH scavenging activity (antioxidant activity) (**a**) and PAL enzyme activity (**b**) of the grape (*Vitis vinifera* L.) cv. Red Sultana. Different letters indicate significantly different values at *p* ≤ 0.05.

The antioxidant capacity was enhanced through the application of all treatments with the highest activity at 10 and 20 mM CTS-SA NPs, introducing the 10 mM concentration as the best (Figure 4a). The CTS-SA NP treatments increased the PAL enzyme activity; the highest enzyme activity was recorded at a 20 mM concentration (Figure 4b).

## 4. Discussion

SA and CTS, individually, were confirmed to have affirmative impacts on plant growth and development and physiological processes through also enhancing the cell number and enlargement and nutrient uptake [2,15,16,24], which could explain that the SA, CTS, and CTS-SA NP treatments had positive effects on the FW and DW in the current study. The SA pre-harvest application (during véraison) enhanced the berries FW and the number of berries per cluster [10]. SA could enhance the membrane permeability and then improve the mineral absorption and assimilate transportation, which consequently leads to FW and DW enhancement [44]; other points confirming the current findings. CTS-SA NPs improved the FW and DW of Moldavian balm plants [24] and the root DW of bitter melon [35] under normal conditions, which is somewhat in line with the current results regarding CTS-SA NP treatments. CTS-SA NPs could contain the CTS and SA individual impacts on plant growth and development even better, thanks to the nano-form and size. It is probable that CTS-SA NPs might enhance the absorption of water and nutrients and improve cell divisions and enlargement. TA, TSS, their ratio, and pH are considered as key factors in fruit quality [7,45]. CTS and CTS-Phe NPs decreased the TA and increased the TSS and TSS/TA values of Flame Seedless grapes, resulting in improved quality of grape via postponement of aging and changing respiration [30]. Hazarika and Marak [46] and Lo’ay and Taher [18] reported a decrease in TA and an increase in the TSS and TSS/TA ratio of grape after SA application during postharvest, in accordance with the current results. On the other hand, Gomes et al. [10] stated no effect of SA treatment on TA and TSS. The differences in the results might be referred to the variety or species and dosage and time of treatment. Most likely, SA may alter the respiration and action of sucrose-phosphate synthase, a key enzyme in sucrose biosynthesis that affects the TA and TSS contents. In fact, SA probably regulated the activities of their synthetic and hydrolytic enzymes. An increase in pH after CTS and the CTS-Phe NP treatments was also reported in grapes [30].

SA could enhance the vit C and sugar content of grape berries, where the higher concentration, the higher the vit C content [46]. The SA application increased the total carbohydrates and carotenoids due to increased carotenoid synthesis [10,47]. The CTS application also enhanced the carotenoids [32]. CTS and CTS-Phe NPs increased the vit C content [30]. The CTS-Se NPs also improved the vit C, total carbohydrates [35], and carotenoids [24,35]. In fact, the enhanced positive effect of CTS-SA NPs on the vit C, total carbohydrates, and carotenoids could be defined through the enhanced effects of SA and CTS in CTS-SA NP form, probably by affecting the involved enzymes or genes in their biosynthesis.

Phenolic compounds and their derivatives, as non-enzymatic and water-soluble antioxidants with potential to detoxify ROS and free radicals, could protect plant cells [30]. Hence, they decrease the risk of many diseases, consequently with beneficial health effects for humans [7,8,45]. Recently, prevalent attention has been dedicated to these beneficial properties on human health, which has not been confirmed by a toxicity report demonstrating their safety, particularly concerning anthocyanins that was verified by a wide consumption of food products containing anthocyanins. An increase in the production of these compounds, predominantly anthocyanins, could cause noteworthy impacts in the marketability of grape berries due to improved color, aside from better nutritional values. The SA effects on the enhancement of phenolic compounds, anthocyanins [2,10,18], phenylpropanoids, flavonols, and the gene expression related to the synthesis of flavonoids [10] were previously confirmed, particularly in grapes. The application of 100 and 200 mM SA at the pre-véraison stage increased the phenolics; 50, 100, and 200 mM SA concentrations (high doses) enhanced the anthocyanin content, particularly oenin, which was twice more at 200 mM, a remarkable increase. The HPLC results also confirmed the results [2]. CTS application enhanced phenolic compounds, flavonoid [32], anthocyanin content [32,33]. A similar positive effect of CTS application on phenolics was reported in raspberry [33]. CTS and CTS-Se NPs enhanced the phenolic compounds [24]. Similarly, CTS and CTS-Phe NPs mostly enhanced the total phenolic compounds, flavonoid and anthocyanin contents of grape. The CS could have an elicitor-like effect in polyphenol biosynthesis [30]. Sheikhalipour et al. [35] reported enhanced anthocyanins after CTS-Se NP application. Phenolic enhancement by CTS-SA NP application could be described via probable roles in their biosynthesis or prevent their degradation, possibly through effects on their genes or enzymes. PAL enzyme activity enhances the production of phenolic compounds, flavonoids, and anthocyanins in different plant tissues, consequently, the higher the PAL activity, the higher the content of phenolic compounds, flavonoids, and anthocyanins, in line with the current findings. In fact, in the present research, the positive effect of SA on the expression and activity of PAL and probably other enzymes related to the biosynthesis of these metabolites increased the phenolic compounds, flavonoids, and particularly, anthocyanins. The SA application enhanced oenin as the dominant anthocyanin in red grapes [2], similar to the current findings. Gomes et al. [10] additionally reported the encouraging effects of SA pre-harvest application (through berry growth and véraison) on the phenolic profile by enhancing malvidin, monoglycosylated cyanidin, and delphinidin. SA probably affects the genes that control the output of this anthocyanin and other compounds in grape aside from PAL. The best results regarding oenin and the other studied compounds by HPLC was achieved by the application of CTS-SA NPs, which confirmed our hypothesis that using nano-form and CTS as a carrier could cause better effects of SA at lower doses with a less toxic effect.

The PAL enzyme plays crucial roles in increasing the production of phenolic compounds in plant tissues, as established earlier. This enzyme is responsible for the (poly) phenolic derivatives through a shift from the primary (shikimate pathway) to the secondary metabolic (phenylpropanoid pathway) pathways [48]. SA acts as an efficient elicitor to prompt PAL activity [2]. SA increased the transcription of the PAL genes [22,49]. CTS also enhanced the PAL enzyme activity [33]. CTS acts as an elicitor in PAL activation and some other enzymes, playing a role in polyphenol biosynthesis. CTS and CTS-Phe NPs enhanced PAL activity in grape [30]. As a result, the increases in the phenolic compounds, flavonoids, and anthocyanins in the present study could be related to the enhanced activity of the PAL enzyme. In addition, the best result was achieved by the application of CTS-SA NPs, representing a better efficiency of the SA impacts in the NP form due to using a carrier and the nano-size confirming the study theory. In addition, PAL enhancement resulted in diversion of the shikimate pathway toward the phenylpropanoid pathway (the synthesis of secondary compounds such as phenolics), leading to the production of many valuable plant metabolites with antioxidant activity [2]. Phenolic compounds, flavonoid and anthocyanins as well as vit C have strong antioxidant activities and a close relationship was realized between them and antioxidant capacity. Accordingly, an increase in their amounts enhances the antioxidant capacity [7,8,45]. SA could enhance antioxidant systems in grapes as confirmed by DPPH method [2,10,50]. Likewise, CTS application enhanced the antioxidant capacity of strawberry [32] and raspberry [33]. CTS and CTS-Phe NPs also increased the antioxidant capacity of grape [30]. Similarly, the CTS-Se NPs enhanced the antioxidant capacity [35]. Consequently, the present findings confirmed the enhanced antioxidant capacity in grape berries after imposing all treatments that could be in line with previous studies regarding SA and CTS and different NPs including CTS. The CTS-SA NPs could contain SA, and CTS predominantly impacts the positive effects of SA on the enhancement of phenolic compounds, flavonoids, anthocyanins, PAL, and then also after the antioxidant capacity of grape berries.

## 5. Conclusions

The best results were mostly obtained for the FW, DW, TS, TSS, 413 pH, vit C, total carbohydrate, carotenoids, total phenolics, flavonoids, antioxidant capacity, and especially the anthocyanin and PAL enzyme activity as well as oenin by the CTS-SA NP treatments and even mostly at the CTS-SA NP lower dose. Therefore, CTS-SA NPs might be an appropriate treatment to enhance the quality and nutritional value of grape berries by enhancing anthocyanins at lower doses of SA to be considered almost safer for human consumption. In addition, the enhancement in anthocyanins, especially oenin, as the dominant anthocyanin of red grapes, increased the visual quality aside from the healthy quality via the increase in nutritional compounds. Hence, spraying grape berries at pre-véraison with CTS-SA NPs could be a convenient strategy for the noticeable increase in phenolic compounds, flavonoids, and particularly anthocyanins, which resulted in the higher quality and nutritional properties of grape.

## Figures and Tables

**Table 1:** The effect of salicylic acid (SA) (10 and 20 mM), chitosan (CTS) (0.1%), and their combination in the nano-form (CTS-SA NPs) treatments on fresh (FW) and dry (DW) weights, titrable acidity (TA), total soluble solids (TSS), TSS/TA ratio, and pH of the grape (*Vitis vinifera* L.) cv. Red Sultana. Different letters indicate significantly different values at *p* ≤ 0.05.

Treatment	Berry FW (g)	Berry DW (g)	TA (%)	TSS (%)	TSS/TA	pH
Control	3.056 ± 0.45 ^c^	0.26 ± 0.031 ^de^	0.386 ± 0.024 ^a^	11.304 ± 1.55 ^e^	29.28 ± 1.24 ^e^	3.62 ± 0.4 ^c^
SA 10 mM	4.108 ± 0.37 ^b^	0.31 ± 0.018 ^bc^	0.334 ± 0.024 ^bc^	13.27 ± 0.88 ^cd^	39.73 ± 2.24 ^c^	4.27 ± 0.1 ^bc^
SA 20 mM	4.566 ± 0.67 ^ab^	0.33 ± 0.034 ^b^	0.323 ± 0.061 ^bc^	15.288 ± 1.31 ^b^	47.32 ± 3.2 ^b^	4.41 ± 0.15 ^b^
CTS NPs 0.1%	4.305 ± 0.41 ^b^	0.28 ± 0.028 ^d^	0.385 ± 0.059 ^a^	13.841 ± 0.95 ^c^	35.95 ± 3.29 ^d^	3.96 ± 0.22 ^b^
CTS-SA NPs 10 mM	4.187 ± 0.28 ^b^	0.39 ± 0.045 ^a^	0.323 ± 0.048 ^bc^	16.46 ± 0.67 ^a^	50.95 ± 3.24 ^a^	4.65 ± 0.38 ^a^
CTS-SA NPs 20 mM	4.891 ± 0.31 ^a^	0.41 ± 0.018 ^a^	0.343 ± 0.036 ^b^	17.183 ± 1.2 ^ab^	50.096 ± 6.2 ^a^	4.77 ± 0.24 ^a^

**Table 2:** The effect of salicylic acid (SA) (10 and 20 mM), chitosan (CTS) (0.1%), and their combination in nano-form (CTS-SA NPs) treatments on some anthocyanins, particularly malvidin-3-*O*-b glucoside (oenin) of grape (*Vitis vinifera* L.) cv. Red Sultana based on the HPLC chromatogram. The anthocyanin was isolated by the solid phase extraction of C-18 cartridges using 5% formic acid and methanol in a linear gradient from 15 to 35% for 15 min, followed by isocratic elution for 20 min. Different letters indicate significantly different values at *p* ≤ 0.05.

Treatments	Control	SA 10 mM	SA 20 mM	CTS NPs 0.1%	CTS-SA NPs 10 mM	CTS-SA NPs 20 mM
Delphinidin-3-O-monoglucoside (mg Mlv g^−1^ FW)	2.45 ± 0.89 ^d^	6.71 ± 0.67 ^c^	7.94 ± 0.91 ^bc^	6.37 ± 0.39 ^c^	8.75 ± 0.42 ^b^	10.89 ± 0.38 ^a^
Cyanidin-3-O-monoglucoside (mg Mlv g^−1^ FW)	0.94 ± 0.12 ^d^	1.59 ± 0.18 ^bc^	1.88 ± 0.18 ^b^	1.07 ± 0.13 ^c^	2.51 ± 0.19 ^a^	2.54 ± 0.27 ^a^
Petunidin-3-O-monoglucoside (mg Mlv g^−1^ FW)	1.81 ± 0.89 ^c^	2.19 ± 0.37 ^b^	3.87 ± 0.68 ^a^	1.47 ± 0.27 ^cd^	2.08 ± 0.6 ^b^	3.08 ± 0.28 ^b^
Peonidin-3-O-monoglucoside (mg Mlv g^−1^ FW)	3.67 ± 0.98 ^d^	4.29 ± 0.67 ^cd^	6.04 ± 0.99 ^b^	4.67 ± 1.48 ^c^	6.98 ± 0.29 ^ab^	7.08 ± 0.84 ^a^
Malvidin-3-O-monoglucoside (Oenin) (mg Mlv g^−1^ FW)	12.41 ± 1.38 ^d^	17.36 ± 2.45 ^c^	19.89 ± 2.84 ^b^	14.25 ± 3.05 ^cd^	21.27 ± 1.95 ^ab^	23.37 ± 2.07 ^a^
Malvidin-3-O-acetylmonoglucoside (mg Mlv g^−1^ FW)	5.23 ± 1.08 ^cd^	6.36 ± 2.36 ^c^	10.91 ± 3.07 ^ab^	8.04 ± 2.59 ^bc^	9.07 ± 1.97 ^b^	12.91 ± 2.19 ^a^
Malvidin-3-(6-O-p-coumaroyl) monoglucoside (mg Mlv g^−1^ FW)	4.04 ± 0.98 ^bc^	8.38 ± 1.38 ^a^	6.45 ± 0.81 ^ab^	5.17 ± 1.04 ^b^	7.08 ± 1.54 ^ab^	8.79 ± 0.87 ^a^

## Data Availability

The data that support the findings of this study are available from the corresponding author upon reasonable request.
